# Characteristics, Outcomes, and Risk Factors in Primary and Secondary Angiosarcoma: A Retrospective Cohort Study

**DOI:** 10.1155/tbj/7158762

**Published:** 2025-10-10

**Authors:** Flemming Puscz, Nilofar Ahmadi, Sonja Verena Schmidt, Felix Reinkemeier, Marius Drysch, Yonca Steubing, Maximilian Völlmecke, Marcus Lehnhardt, Christoph Wallner

**Affiliations:** ^1^Department of Plastic Surgery and Hand Surgery, Burn Centre, Sarcoma Centre, BG University Hospital Bergmannsheil, Ruhr University Bochum, Bochum, Germany; ^2^Department of Trauma Surgery and Orthopaedics, Reconstructive and Hand Surgery, Burn Medicine, German Armed Forces Central Hospital Koblenz, Koblenz, Germany

## Abstract

**Background:**

Angiosarcomas (ASs) are a heterogeneous subtype of soft tissue sarcomas. They can be subdivided into primary and secondary AS, with secondary AS being predominant, particularly those following radiotherapy. The aim of this study was first to analyze our patient cohort on a descriptive level and then to identify possible risk factors with regard to one and 5-year survival using logistic regression.

**Methods:**

The study was designed as a retrospective, single-center cohort study. All patients with histologically confirmed AS over 18 years of age were included in the study. Binary logistic regression was used for univariate analysis screening of continuous or dichotomous variables, respectively. For multivariate analysis, binary multivariate logistic regression was performed to assess independent associations between chosen variables and AS.

**Results:**

A total of 39 patients were included in this study. 14 (35.9%) had primary and 25 (64%) had secondary AS. Women were more frequently affected (76.9%) than men (23.1%). The 1-year survival rate was 87.2%, and the 5-year survival rate was 51.3%. In the logistic regression analyses, nicotine consumption and a history of carcinoma were identified as significant factors influencing the 1-year survival rate. For the 5-year survival rate, only breast cancer was found to be a significant influencing factor in the univariate analysis. Based on univariate logistic regression, all variables with a *p* value of < 0.1 were chosen to be included into multivariate analysis. The multivariate analysis showed diabetes mellitus (*p*=0.067) with an association to influence the 5-year survival rate.

**Conclusions:**

We were able to show that the proportion of secondary ASs is predominant. These occur after radiation treatment of the breast. Diabetes mellitus may be associated with reduced 5-year survival, although this finding did not reach statistical significance and requires further investigation. Due to its small sample size, this study should be regarded more as hypothesis-generating.

## 1. Introduction

Angiosarcomas (ASs) are rare malignancies with an incidence of 3.3 cases per 1,000,000 person-years [1]. Approx. 2% of all soft tissue sarcomas can be assigned to this entity [2]. They can be divided into primary AS, i.e., spontaneously occurring, and secondary AS, due to a previous illness or condition. Radiation is known to be a major risk factor for the development of secondary AS [3]. Since radiation is one of the main treatment options in breast cancer, particularly in breast-conserving therapy [4], special attention must be paid to secondary ASs of the breast. These occur with a frequency of 1 in 1000 patients in 10 years [5] and have been on the rise in recent years due to the increase in breast-conserving therapy for invasive breast carcinoma or ductal carcinoma in situ [6]. This increasing burden poses a considerable clinical challenge, which must be addressed. Patients already exposed to the physical and psychological impact of breast cancer therapy face an additional, aggressive malignancy, which markedly affects long-term prognosis [7]. Current literature therefore recommends extended follow-up for more than 20 years after breast cancer treatment [5]. The true magnitude of secondary AS incidence and its clinical implications remain debated as it is unclear whether the observed increase reflects genuine epidemiological changes or merely evolving histopathological definitions and improved diagnostic awareness [1]. Recent population-based studies support the notion of a rising incidence. A large US registry analysis reported that the number of newly diagnosed AS cases doubled from 657 in 2001 to 1312 in 2019, corresponding to an average annual increase of 1.6% and a current incidence of approximately 3.3 cases per million person-years [1]. Similarly, the Surveillance, Epidemiology, and End Results (SEER) data spanning 1975–2016 revealed a significant increase in age-adjusted incidence from 0.13 to 0.33 per 100,000 individuals, with an annual percentage change of 2.4% [8]. Particularly striking was the rise among patients with a prior malignancy, where the incidence of secondary AS increased from 0.03 to 2.25 per 100,000 between 1973 and 2014 [9]. Whether this represents a true epidemiological increase or improved diagnostic awareness remains unclear, but the growing number of cases highlights the importance of detailed clinical and epidemiological characterization. Against this background, the present study analyzes a single-center cohort of patients with histologically confirmed AS to compare institutional observations with reported epidemiological trends, describe differences in demographics, comorbidities, management, and outcomes between primary and secondary AS, and explore potential risk factors associated with survival.

## 2. Materials and Methods

This study is a retrospective, single-center cohort study conducted in compliance with the principles outlined in the Declaration of Helsinki. Ethical approval was obtained from the Ethics Committee of the Ruhr-University Bochum (reference number: 20-7067_1-bio). Written informed consent was acquired from all participants for the use and publication of this paper. The report adheres to the “Strengthening the Reporting of Observational Studies in Epidemiology” (STROBE) guidelines for cohort studies.

### 2.1. Participants

All patients over 18 years of age who underwent sarcoma surgery in our clinic between 2001 and 2019 were screened for this study. Inclusion criteria included a histologically confirmed AS according to the World Health Organization's (WHO) definition of soft tissue sarcomas from 2020 [10]. Comorbidities were recorded during the initial medical history interview. Patients with missing data and/or incomplete medical records were excluded from the study. Patients who underwent primary surgery or neoadjuvant therapy elsewhere were not excluded if the data were complete.

### 2.2. Outcome Assessment and Data Collection

The primary study endpoint was 5-year survival. As sarcoma patients come to our clinic at least once a year for a follow-up examination, this endpoint could be tracked reliably. The patients were identified from the clinic's internal sarcoma database. The study team was then able to transfer the necessary patient data from the hospital information system to a separate data collection. All data were double-checked by two persons trained in the study protocol.

### 2.3. Pre-, Intra-, and Postoperative Management

Patients present to our clinic for the first time with a suspected sarcoma or with an already prediagnosed AS. Clinically, AS may appear as a dark-colored hematoma or nodule, which later develops into an ulceration [11]. It is therefore important to clarify these initial abnormalities in patients with known risk factors. MRI and CT examinations can already provide an indication of the presence of AS, but biopsy (either punch or incisional) remains the method of choice for a reliable diagnosis [12]. However, a punch biopsy can prove difficult, especially in the breast, due to the extensive tumor growth. Vacuum-assisted punch biopsy can also be advantageous in these cases [13]. Once the staging is complete, the further treatment is discussed in the interdisciplinary tumor conference and usually the indication for surgery is made. Regarding the resection margins, a radical approach has become established, particularly for radiation-associated ASs. This involves the resection of the entire former radiation field and not just the areas affected by the tumor. This procedure shows a lower recurrence rate and improved 5-year survival [6]. After the R0 status has been confirmed by pathology and the case is presented again at the tumor conference, adjuvant radio- or/and chemotherapy is usually indicated, depending on the grade of the tumor. Patients are then closely linked to our clinic for follow-up care and undergo a follow-up examination (usually chest X-ray and contrast-enhanced MRI of the former tumor area) every 3 months for the first 2 years. For a further 3 years, they undergo follow-up assessments every 6 months and then annually.

### 2.4. Choice of Candidate Variables

To identify potential variables for analysis, all patient data, diagnoses, and associated organ dysfunction variables available in our database were considered. Initially, these variables were assessed through univariate analysis. Given that 19 events were observed in this study, a maximum of two covariates could be included in the multivariate analysis [14]. Only variables with a *p*value < 0.1 in the univariate analysis and a meaningful association with AS were included in the model. This threshold was chosen to minimize the risk of excluding potentially relevant predictors in this small cohort. Given the limited number of events, the number of covariates was restricted to avoid model overfitting, and the results should therefore be interpreted as exploratory and hypothesis-generating.

### 2.5. Statistical Analysis

Data were collected using Microsoft Excel Version 16.84. Statistical analyses were performed using IBM SPSS Version 25 and Prism Version 10.3.1. Continuous variables are presented as means with standard deviation and 95% confidence intervals. Categorical variables are presented as counts, percentages, and corresponding 95% confidence intervals, where appropriate. Fisher's exact test was used to compare categorical variables for descriptive statistics. A Shapiro–Wilk test was first carried out to select the appropriate statistical test for the continuous variables. Significance testing was then performed using a *t*-test or Mann–Whitney-U-test. Binary logistic regression was used for univariate analysis screening of continuous or dichotomous variables, respectively. Based on univariate logistic regression, all variables with a *p* value of < 0.1 were chosen to be included into multivariate analysis for 5-year survival. As there were only 5 events at 1-year survival, no meaningful multivariate analysis could be performed here. For multivariate analysis, binary multivariate logistic regression was performed to assess independent associations between chosen variables and AS. A *p* value of < 0.05 was considered as significant.

## 3. Results

### 3.1. Study Cohort

After reviewing the hospital's internal sarcoma database, a total of 49 patients with histologically confirmed AS were identified. Of these, 39 patients could be included in the further data analysis, as the data were incomplete for 10 patients. A total of 14 patients (35.9%) with primary and 25 patients (64%) with secondary AS were included. The mean age at diagnosis was 66 years. Women were more frequently affected (76.9%) than men (23.1%). The 1-year survival rate was 87.2%, and the 5-year survival rate was 51.3%.  Table 1 shows the detailed characteristics. ASs are usually high-grade sarcomas but there is no standardized grading which was therefore not included in the evaluation [10].

### 3.2. Univariate Analysis for 1-Year and 5-Year Survival

From this dataset, we assessed all possible variables in univariate analysis for 1-year and 5-year survival. Nicotine consumption (OR 0.043, 95% CI: 0.004–0.469, *p*=0.01) and another carcinoma in the patient's history (OR 0.065, 95% CI: 0.008–0.552, *p*=0.012) were significantly associated with a worse 1-survival (see Table 2).

For 5-year survival, only breast cancer (OR 5.143, 95% CI: 1.299–20.360, *p*=0.02) showed significance in affecting the outcome of AS (see Table 3). Although not statistically significant, diabetes mellitus (*p*=0.061) and female sex (*p*=0.061) showed a trend toward association with reduced 5-year survival.

### 3.3. Multivariate Analysis for 5-Year Survival

Based on our findings from the univariate analysis, all variables with a *p* value < 0.1 from 5-year survival were included in a multivariate binary logistic regression. The following variables were included: breast cancer, female sex, and diabetes mellitus (see Table 4). The presence of diabetes mellitus was associated with a decreased odds of 5-year survival (OR: 0.178, 95% CI: 0.028–1.13, *p*=0.067), approaching significance with *p*=0.067. Neither breast cancer (OR: 2.99, 95% CI: 0.493–18.121, *p*=0.233) nor gender (OR: 0.356, 95% CI: 0.038–3.339, *p*=0.366) showed a statistically significant association with 5-year survival after adjustment for confounders.

## 4. Discussion

In this study, we could demonstrate the characteristics and outcomes of primary and secondary AS in our patient cohort. Our analysis identified approaching significance between diabetes mellitus and reduced 5-year survival in patients with AS, while no significant associations were observed for prior history of mamma carcinoma.

### 4.1. Epidemiology and Risk Factors

ASs are usually sarcomas of the elderly. According to this large epidemiological study using SEER data by Albores-Saavedra and colleagues [15], the average age of onset is 73 years. In our cohort, the patients were slightly younger, with an average of 66 years. This discrepancy might be attributed to the inclusion of secondary AS in our analysis, as these often develop earlier following therapeutic interventions such as radiation. According to this study [15], the gender distribution in AS patients is almost evenly distributed. In our data, however, women (76.9%) were more frequently affected than men. This is mainly since 24 secondary ASs were included in our total of 39 patients.

The fact that secondary AS almost exclusively affects women is also consistent with data from previous studies [16]. This is likely due to the high prevalence of breast cancer treatments involving radiotherapy. Irradiation remains the greatest risk factor for the development of secondary AS [17, 18]. Interestingly, this pattern underscores the importance of long-term monitoring in patients undergoing radiotherapy, particularly women treated for breast cancer. Other preexisting conditions, such as chronic lymphedema, are much less frequently associated with the development of secondary AS (Stewart–Treves syndrome) but should not be disregarded [19]. In addition to the significant effect of breast cancer history in the univariate analysis, variables such as diabetes mellitus and female sex demonstrated trends toward an association with 5-year survival. While these observations did not reach statistical significance (both *p*=0.061), they may represent clinically meaningful patterns deserving further study.

UV exposure [20, 21], exogenous toxins (e.g., arsenic) [22, 23], and syndromic diseases (e.g., neurofibromatosis type 1 [24]) are also associated with the development of secondary AS. These risk factors underscore the need for a detailed patient history and consideration of environmental and genetic predispositions when assessing AS etiology. In our cohort, 24 of 25 secondary ASs were cutaneous AS of the breast after radiation. The only male patient with a secondary AS developed this after radiotherapy to the upper extremity due to a liposarcoma.

It is also noteworthy that obesity does not appear to have any relevant influence on the 1-year and 5-year survival rates, which is in line with the data from this large meta-analysis [25]. In terms of survival rates, our data with a 5-year survival rate of 51.3% are slightly better than the large datasets from previous studies [26, 27]. This improvement might reflect advancements in multimodal treatment approaches, including optimized surgical techniques, radiotherapy protocols, and systemic therapies.

### 4.2. Impact of Diabetes Mellitus on 5-Year Survival

First of all, it must be emphasized that the results of the multivariate analysis only represent a potential association of diabetes mellitus on 5-year survival. Statistical significance could not be achieved. Nevertheless, this association should be compared with the results from previous research. Given the rarity of ASs, the identification of potential prognostic factors such as diabetes mellitus is particularly crucial, as it may guide the limited therapeutic options available for this patient population. The observed trend for diabetes mellitus as a risk factor aligns with prior studies highlighting the role of metabolic comorbidities in cancer prognosis [28–30]. Diabetes may not only appear to be associated with poorer outcomes in cancer but, as recent studies suggest, it may also play a role in the development of certain malignancies [31]. Interestingly, this large cohort study identified diabetes mellitus, along with hypertension, as a risk factor for the development of AS [32]. Diabetes mellitus may therefore represent a potential factor influencing both the development and outcome of the disease. However, given the lack of statistical significance in our cohort, this observation should be regarded as exploratory and hypothesis-generating, requiring confirmation in larger prospective studies. The reasons for the influence of diabetes on the outcome are multifactorial: it may contribute to poorer survival due to chronic inflammation [33], hyperglycemia-induced oxidative stress [34], and its impact on immune surveillance, mechanisms that are well documented in cancer progression [35]. Furthermore, the interplay between diabetes and cancer therapies, such as chemotherapy or radiotherapy, might exacerbate these effects, potentially compromising treatment efficacy. Despite its likely relevance, the role of diabetes mellitus in AS patients has not yet been investigated in detail. Existing literature on this topic is limited to case reports that briefly describe the presence of diabetes in AS patients [36]. This study should therefore serve as a possible impetus for further investigations, in particular prospective studies with the inclusion of HbA1c values should be carried out here. If confirmed in larger cohorts, the observed association between diabetes mellitus and survival may underscore the need for metabolic risk factor management in AS patients.

### 4.3. Limitations

This study has several limitations that warrant consideration. First, the relatively small sample size of 39 patients limits the generalizability and statistical power of our findings. In particular, the fact that we were unable to find any significant results in the multivariate analysis must be emphasized here. While this cohort represents a substantial single-center dataset for AS, the sample size constrains our ability to make definitive conclusions or identify additional risk factors influencing survival in AS patients. Larger, multicenter studies are needed to validate our results and uncover more robust associations.

Another limitation of our analysis is the risk of overfitting in the multivariate model due to the small number of events. Although we restricted the number of covariates according to established recommendations and applied a predefined inclusion threshold (*p* < 0.1), the limited sample size inevitably constrains the robustness of the multivariate results. Consequently, our findings should be regarded as exploratory and primarily hypothesis-generating, requiring confirmation in larger, prospective cohorts.

In addition, some of the logistic regression models yielded extremely large odds ratios with infinite confidence intervals. This is a statistical artifact that arises from the small sample size and zero events in certain subgroups, leading to quasi-complete separation in the model. These estimates should therefore not be interpreted as clinically meaningful but rather illustrate the inherent limitations of regression analysis in very small datasets.

Furthermore, as this was a retrospective analysis, our data were limited to the information available in hospital records. This entails the risk of incomplete documentation and missing variables, which may have introduced bias. Potential misclassification also cannot be excluded, for example, regarding comorbidities or treatment history, where diagnostic accuracy and coding practices may have varied over time. Regarding diabetes mellitus, we were unable to differentiate between type 1, type 2, and other forms of diabetes within our dataset. Additionally, the absence of HbA1c values precludes any conclusions about glycemia or patient compliance, which may be relevant factors influencing outcomes.

Therefore, our study's findings should be interpreted as hypothesis-generating rather than definitive. Prospective studies with larger cohorts and comprehensive data collection are necessary to confirm and expand upon our observations. Particular emphasis should be placed on standardized assessment of comorbidities and metabolic parameters, including glycemic control markers such as HbA1c, to better clarify the role of diabetes mellitus and related conditions in AS prognosis. In addition, molecular and genetic profiling may help identify subgroups with distinct risk profiles and therapeutic vulnerabilities. These efforts are essential to advance personalized management strategies and to improve outcomes in this rare and aggressive disease.

## 5. Conclusions

We were able to show that our demographics and outcome data correlate with the existing findings from preliminary studies. The fact that more women appear to be affected in our cohort is not consistent with the preliminary studies which is most likely due to our cohort size and the preponderance of secondary AS after breast cancer underlining the importance of this patient group. Regarding potential prognostic factors, diabetes mellitus showed a trend toward an association with worse 5-year overall survival; however, statistical significance was not achieved. This observation should therefore be regarded as exploratory and hypothesis-generating. Larger, prospective studies with comprehensive clinical and metabolic data are needed to confirm or refute this potential association and to guide future clinical management.

## Figures and Tables

**Table 1:** Patient characteristics.

	All AS patients (*N* = 39)	All AS 95% CI	Patients with primary AS (*N* = 14)	Primary AS 95% CI	Patients with secondary AS (*N* = 25)	Secondary AS 95% CI	*p* value
Baseline characteristics							
Mean age at initial diagnosis (years)	66 ± 14	61.5–70.5	60 ± 17	50.2–69.8	70 ± 11	65.5–74.5	0.08
Female sex (%)	30 (76.9)	61.7%–87.4%	6 (42.9)	21.4%–67.4%	24 (96)	80.5%–99.3%	0.0004
Body mass index (kg/m^2^)	27.3 ± 5.1	25.6–29.0	28 ± 4.8	25.2–30.8	27 ± 5.4	24.8–29.2	0.81
Comorbidities (%)							
Hypertension	25 (64.1)	48.4%–77.3%	6 (42.9)	21.4%–67.4%	19 (76)	56.6%–88.5%	0.08
Diabetes mellitus	9 (23.1)	12.6%–38.3%	3 (21.4)	7.6%–47.6%	6 (24)	11.5%–43.4%	> 0.9
Obesity	9 (23.1)	12.6%–38.3%	4 (28.6)	11.7%–54.6%	5 (20)	8.9%–39.1%	0.7
Breast cancer	22 (56.4)	41.0%–70.7%	0 (0)	0.0%–21.5%	22 (88)	70.0%–95.8%	< 0.0001
Other carcinoma	6 (15.4)	7.2%–29.7%	3 (21.4)	7.6%–47.6%	3 (12)	4.2%–30.0%	0.65
History of chemotherapy	9 (23.1)	12.6%–38.3%	0 (0)	0.0%–21.5%	9 (36)	20.2%–55.5%	0.015
History of radiotherapy	25 (64.1)	48.4%–77.3%	0 (0)	0.0%–21.5%	25 (100)	86.7%–100.0%	< 0.0001
Nicotine	9 (23.1)	12.6%–38.3%	3 (21.4)	7.6%–47.6%	6 (24)	11.5%–43.4%	> 0.9
Tumor characteristics (%)							
Metastasis	11 (28.2)	16.5%–43.8%	5 (35.7)	16.3%–61.2%	6 (24)	11.5%–43.4%	0.48
Recurrence	22 (56.4)	41.0%–70.7%	4 (28.6)	11.7%–54.6%	18 (72)	52.4%–85.7%	0.017
Treatment (%)							
Complete resection	26 (66.7)	51.0%–79.4%	9 (64.3)	38.8%–83.7%	18 (72)	52.4%–85.7%	0.72
Adjuvant radiotherapy	11 (28.2)	16.5%–43.8%	4 (28.6)	11.7%–54.6%	7 (28)	14.3%–47.6%	> 0.9
Adjuvant chemotherapy	5 (12.8)	5.6%–26.7%	3 (21.4)	7.6%–47.6%	2 (8)	2.2%–25.0%	0.33
One-year survival rate	34 (87.2)	73.3%–94.4%	11 (78.6)	52.4%–92.4%	23 (92)	75.0%–97.8%	0.33
Five-year survival rate	20 (51.3)	36.2%–66.1%	5 (35.7)	16.3%–61.2%	15 (60)	40.7%–76.6%	0.19

*Note:* AS = angiosarcoma.

**Table 2:** Univariate logistic regression for all variables associated with 1-year survival.

Variables	Odds ratio	95% CI	*p* value
Nicotine	0.043	0.004–0.469	0.010
Other carcinoma	0.065	0.008–0.552	0.012
History of radiotherapy	3.136	0.456–21.566	0.245
Female sex	0.389	0.054–2.797	0.348
Recurrence	2.308	0.338–15.75	0.393
Breast cancer	2.143	0.316–14.545	0.435
Hypertension	0.404	0.041–4.020	0.439
Complete resection	2.182	0.218–21.793	0.506
Adjuvant radiotherapy	1.667	0.165–16.827	0.665
Age at initial diagnosis	0.985	0.914–1.063	0.704
Diabetes mellitus	1.231	0.120–12.652	0.861
History of chemotherapy	1.231	0.120–12.652	0.861
Obesity	1.231	0.120–12.652	0.861
Adjuvant chemotherapy	119,175,544	0–∞	0.999
Metastasis	13,269,000,000,000	0–∞	0.999

*Note:* Extremely large odds ratios with infinite confidence intervals result from sparse data and quasi-complete separation in the logistic regression model.

Abbreviation: CI = confidence interval.

**Table 3:** Univariate logistic regression for all variables associated with 5-year survival.

Variables	Odds ratio	95% CI	*p* value
Breast cancer	5.143	1.299–20.360	0.02
Female sex	0.19	0.034–1.078	0.061
Diabetes mellitus	0.19	0.034–1.078	0.061
History of radiation	2.7	0.697–10.465	0.151
Obesity	0.382	0.080–1.825	0.228
Hypertension	0.437	0.113–1.681	0.228
Complete resection	1.867	0.480–7.255	0.368
Recurrence	0.583	0.162–2.097	0.409
Nicotine	0.7	0.157–3.130	0.641
History of chemotherapy	0.7	0.157–3.130	0.641
Metastasis	0.722	0.178–2.929	0.649
Adjuvant chemotherapy	1.5	0.222–10.143	0.678
Age at initial diagnosis	1.009	0.963–1.056	0.718
Adjuvant radiotherapy	1.2	0.296–4.862	0.798
Other carcinoma	0	0–∞	0.999

*Note:* Extremely large odds ratios with infinite confidence intervals result from sparse data and quasi-complete separation in the logistic regression model.

Abbreviation: CI = confidence interval.

**Table 4:** Multivariate logistic regression for breast cancer, female sex, and diabetes mellitus associated with 5-year survival.

Variables	Regression coefficient	Standard error	Odds ratio	95% CI	*p* value
Breast cancer	1.095	0.919	2.99	0.493–18.121	0.233
Female sex	−1.033	1.142	0.356	0.038–3.339	0.366
Diabetes mellitus	−1.728	0.944	0.178	0.028–1.13	0.067

Abbreviation: CI = confidence interval.

## Data Availability

The data that support the findings of this study are available from the corresponding author upon reasonable request.
